# Adaptive Immunity: New Aspects of Pathogenesis Underlying Neurodegeneration in Glaucoma and Optic Neuropathy

**DOI:** 10.3389/fimmu.2020.00065

**Published:** 2020-02-13

**Authors:** Shuhong Jiang, Marie Kametani, Dong Feng Chen

**Affiliations:** ^1^Schepens Eye Research Institute of Massachusetts Eye and Ear, Department of Ophthalmology, Harvard Medical School, Boston, MA, United States; ^2^State Key Laboratory of Ophthalmology, Zhongshan Ophthalmic Center, Sun Yat-sen University, Guangzhou, China

**Keywords:** glaucoma, optic neuropathy, heat shock proteins, T cells, glial response, neuroinflammation

## Abstract

Glaucoma is a globally unmet medical challenge and the most prevalent neurodegenerative disease, which permanently damages the optic nerve and retinal ganglion cells (RGCs), leading to irreversible blindness. Present therapies target solely at lowering intraocular ocular pressure (IOP), a major risk factor of the disease; however, elevated IOP is neither necessary nor sufficient to cause glaucoma. Glaucomatous RGC and nerve fiber loss also occur in individuals with normal IOP. Recent studies have provided evidence indicating a link between elevated IOP and T cell-mediated autoimmune responses, particularly that are specific to heat shock proteins (HSPs), underlying the pathogenesis of neurodegeneration in glaucoma. Reactive glial responses and low-grade inflammation may initially represent an adaptive reaction of the retina to primary stress stimuli; whereas, sustained and excessive glial reactions lead to expanded immune responses, including adaptive immunity, that contribute to progressive neural damage in glaucoma. Emerging data suggest a similar mechanism in play in causing neurodegeneration of other forms of optic neuropathy, such as that resulted from acute ischemia and traumatic injuries. These studies may lead to the paradigm shift and offer a new basis for the development of novel mechanism-based diagnosis, therapy, and preventive interventions for glaucoma. As HSPs are induced under various conditions of neural stress and damage in the brain and spinal cord, these findings may have broader implications for our elucidating of the etiology of other neurodegenerative disorders in the central nervous system.

## Introduction

Glaucoma, which leads to progressive and irreversible vision loss, presents a critical medical challenge, partly due to the poorly studied mechanisms that damage the optic nerve and lead to the death of retinal ganglioncells (RGCs). While current therapy targets solely on lowering the intraocular pressure (IOP), studies have indicated that the pathogenesis of the disease is multifactorial (1). Factors such as genetics, age, and immunity are shown to be critical contributors. In addition, glaucoma can occur in patients with normal IOP, so called normal tension glaucoma (NTG). The therapeutic approach that aims to lower the IOP is insufficient to halt, sometime limited slowing down of the progression of the disease (2). There is strong evidence supported by both experimental and clinical research that elevated IOP triggers secondary responses which are responsible for RGC degeneration in glaucoma. In this review, we examine the evidence regarding the involvement of innate and adaptive immunities, including the induction of T and B cell-mediated responses and microglial activation in the acquisition of pathogenicity in glaucoma. Furthermore, the involvement of heat-shock proteins (HSPs), such as HSP27 and HSP60, both as neuroprotective and degenerative roles in the disease progression is discussed.

## The Eye as an Immune Privileged Site and the Case of Glaucoma

The eye is recognized as an immune privileged site. Immune regulation in the eye is characterized by its active local immunosuppression, which is achieved, in part, through the blood-aqueous and blood-retina barriers, the unique feature of the pigment epithelial cells, and the local production of immunosuppressive cytokines and neuropeptides (3, 4). Immune cells that enter the eye in response to infection or injury usually are induced to undergo apoptosis via the activation of Fas-FasL signaling without causing inflammation or tissue damage (5). Antigenic material being introduced into the eye elicits immune deviation or suppression of T cell-mediated immunity that leads to peripheral immune tolerance to the antigens, a mechanism termed Anterior Chamber Associated Immune Deviation (6). Such immune privilege is thought to protect the retina, which has limited ability for regeneration and self-repair, from the damaging effects of an uncontrolled immune responses (7).

The eye's immune privileged status, however, is affected by diseased conditions, such as in the case of glaucoma, when the blood-retina barrier is compromised and cytokine productions are often altered (8). Activation of both innate and adaptive immune responses is evident in glaucoma. While systemic immune responses to the retina is strictly controlled, residential glial cells, including microglia, astrocytes, and Müller cells, play the roles of immune surveillance in the retina. They are found to become activated in the early stage of glaucoma (9). Recent evidence also reveals the critical involvement of systemic adaptive immune responses in the pathogenesis of glaucoma. Complex patterns of retinal proteins and autoantibodies against retinal specific antigens have been detected in the sera of patients with glaucoma (10, 11). Such patterns of serum protein and antibody profiles may be used for early diagnosis and detection of glaucoma and/or assessment of the progression of the disease (11–13). Further supporting a compromised blood-retinal barrier in glaucoma, autoantibodies were found to have access to the retina, and infiltration of inflammatory leukocytes and macrophages were noted preceding clinical symptoms of glaucoma (14, 15). Increased expression of matrix metalloproteinase in the astrocytes of the optic nerve head (ONH) is thought to associate with the loss of the retinal immune privilege, thus antibody penetration into the eye (16, 17). To date, elucidating the exact involvement of innate and adaptive immunity in pathogenesis of glaucoma remains the key to understanding of the disease etiology (18).

## Innate Immune Responses in Glaucoma

The innate immunity is body's first line of defense against foreign organisms, and it offers a quick response that does not confer long-lasting protection against the same pathogen. In glaucoma, it is elevated IOP, not necessarily a foreign antigen, that triggers an innate immune response, which usually involves resident immune cells, such as microglia, as well as the infiltration of macrophages/monocytes (9). Neuroinflammatory responses generated by microglia are thought to play a leading role in glaucomatous pathophysiology. Studies show that glial activations occur at the early stage of the disease in glaucoma patients and animal models (19–21). Increased microglial activity, cell density, and their expression of complement C1q were noted in the retina and optic nerve in experimental model of glaucoma before RGC and axonal loss is observed (22, 23) and thought to be detrimental to retinal neurons. Support for this notion was the elevation of TNFα in the aqueous humor of glaucoma patients and rodent models of glaucoma, correlating with the worsening of RGC loss (24, 25). Suppression of microglial activation with minocycline or neutralization of TNFα protected RGCs from elevated IOP-induced cell death in rodents (26, 27).

Astrocytes and Müller glia, together called astroglia, also respond to the elevated IOP by developing reactive gliosis, which is characterized by the upregulation of glial fibrillary acidic protein and releasing of chemokines and cytokines, including TNFα. This response is also believed to be a pathological element that contributes to neural damage in glaucoma. More recently, studies revealed that reactive gliosis and TNFα production could be neuroprotective to RGCs. The timing of TNFα expression appears to be crucial as that the early induction of TNFα correlates with RGC survival, while longer-term expression causes RGC degeneration (28). In addition, reactive glial cells produce a variety of neuroprotective molecules, such as insulin-like growth factor-1 (IGF1), to protect RGCs from neural damage (29). Overall, the available evidence strongly suggests that reactive glia are involved in the pathology of glaucoma. To date, the key inflammatory signals that lead to glaucomatous neurodegeneration remain unknown.

## Involvement of T Cells in Glaucoma

Activation of the innate immune system initiates and directs the adaptive immune responses, which involve T and B lymphocytes and are also featured in the glaucomatous pathogenesis. In contrast to the innate immune system, the adaptive immune system requires up to 7 days to be activated. Recent report on the involvement of heat shock protein (HSP)-specific T cells offers the key evidence support a role for autoimmunity in glaucoma (30). However, analysis of T cells in patients and experimental models of glaucoma remains limited; there are only few studies focusing on the presence of specific T lymphocyte subsets in the sera of glaucoma patients. In part, this is because pathogenic antigens in the retina are long thought to induce T regulatory (Treg) cells through a mechanism of ACAID, therefore maintaining the ocular immune privilege. Recent studies of the peripheral blood of glaucoma patients revealed that the immunity activated in glaucoma may not be counterbalanced by an efficient immune suppression (30, 31). Patients with glaucoma exhibited a trend of decreased frequency of Treg and their CD4+ T cells presented a greater stimulation response characterized by increased proliferation and proinflammatory cytokine secretion. Elevated frequencies of CD3^+^CD8^+^ lymphocytes in both patients with NTG and primary open-angled glaucoma were noted, and CD8^+^HLA-DR^+^ lymphocytes were particularly prevalent in NTG. This was accompanied by the increased expre ssion of the soluble interleukin-2 (IL-2) receptor, a marker of T cell activation (32, 33).

The pathogenic role of T cells in glaucomatous neurodegeneration is supported by the evidence that adoptive transfer of T cells from glaucomatous mice results in a progressive loss of RGCs and their axons in recipient mice with a normal IOP (34). A link between elevated IOP and induction of anti-HSP autoimmunity has also been suggested (14, 35). HSP27 and HSP60 immunization in rats induced a pattern of RGC and axon degeneration similar to that was seen in patients with glaucoma (36). A transient infiltration of T cells in the retina was noted 2 weeks after the immunization. *In vitro* study further demonstrated T cell activation following HSP-immunization, which initiated the production of inflammatory cytokine and FasL, resulting in RGC apoptosis. Recently, using mice deficient in CD4^+^ αβ T cells, it was reported that CD4^+^ T cells play a crucial role in propagating RGC degeneration, particularly during the prolonged period of progressive neural damage, in glaucoma (30). Uncovering the association between T cell-mediated autoimmunity and progressive neuron loss in glaucoma may allow the development of novel therapeutic interventions that eventually offer a cure for the disease.

## HSPs as Pathogenic Autoantigens in T Cell-Mediated Glaucomatous Neurodegeneration

Adaptive immune responses are elicited by specific antigen stimulation, and HSPs have been identified as pathogenic autoantigens which evoke T cell responses in glaucoma (30). The stress response is a highly conserved mechanism of cellular responses to a wide variety of physiological challenges (37). The response is characterized by the induction of specific cellular proteins with protective functions, such as HSPs. Intracellular HSPs function as molecular chaperones to prevent protein aggregation and facilitate refolding of dysfunctional proteins, which is critical to the survival of all organisms (38). They are some most abundant intracellular proteins that protect cells from destruction and facilitate neural repair through astrocyte and microglial recruitment in the CNS. However, evidence reveals that their release into the extracellular environment is an indication of loss of cellular integrity, thus acts as “danger signals” and elicit both the innate and adaptive immune responses (39). Extracellular HSPs may activate microglia to secrete pro-inflammatory cytokines, such as IL-1β, IL-6, and TNF-α and trigger innate immune responses through toll like receptor 2 (TLR2) and TLR4 (40–42). They can also be processed and presented by antigen presenting cells to stimulate T cell responses. Continued and prolonged IOP elevation leads to HSP upregulation, autoantibody formation, and immune responses in glaucomatous eyes. Overexpression of HSP27 in neurons exacerbated RGC loss following IOP elevation without affecting RGC numbers under a normal IOP (30). These data suggest that elevated IOP not only upregulates HSPs, but also triggers their extracellular release and evoke immune responses.

Tezel et al. have demonstrated increased immunostaining of HSPs in the glaucomatous eyes (43). In a laser-induced rat and non-human primate models of glaucoma, elevated IOP induced expression of HSP27 and HSP70 (44–47). This elevation of HSPs following increased IOP is suggested to be neuroprotective, due in part to the study investigating the effects of geranylgeranylacetone, an HSP70 inducer developed as an anti-ulcer drug. Geranylgeranylacetone decreased elevated IOP-induced neuronal damage by reducing RGC apoptosis and axon loss (44). Subsequent studies by Wax et al. reported that immunization of rats with HSP27 or HSP60 induced significant RGC loss that was in conformity with the RGC- and nerve bundle-specific lesions observed in patients with NTG (36). Recent studies further demonstrate HSP-specific T cell responses in patients with glaucoma (30). Remarkably, mice raised in the absence of commensal microflora (germ-free mice) did not harbor HSP-specific T cells nor did they develop glaucomatous neurodegeneration after IOP elevation (30). These results are in line with the clinical observation that IOP is neither necessary nor sufficient for glaucomatous neuronal damage. It is the subsequent stress-induced events involving retinal inflammation and T cell-mediated responses that are keys to the pathogenesis of glaucoma. Taken together, family of HSPs are critical modulators of both the homeostatic and cytoprotective as well as pathogenic immune response and neurodegenerative arms of the retina and are thus integral to our understanding of neurodegeneration in glaucoma. Identification of key pathogenic autoantigens associated with glaucomatous T cell responses may also provide a foundation for future exploration of tolerance-based clinical intervention for preventing or treating the disease.

## Concluding Remarks

**Figure 1:**
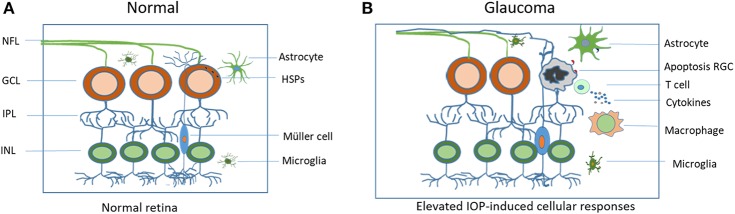
Schematic illustration of elevated IOP-induced cellular responses in the retina. **(A)** Residential glial cells maintain cellular homeostasis in the normal retina. **(B)** In the glaucomatous retina, elevated IOP upregulates heat shock proteins (HSPs), initiates both the innate and adaptive immune responses that include microglial activation and T cell infiltration. Sustained and excessive immune responses lead to RGC apoptosis and vision loss. IOP, intraocular pressure; RGC, retinal ganglion cell; NFL, retinal nerve fiber layer.

Glaucoma is a globally unmet medical challenge due to its prevalence and debilitating consequences. The lack of cure for such a major disease reflects poor understanding of the disease's mechanisms. Numerous clinical and experimental data are now pointing to an unexpected interaction among elevated IOP, HSP-specific T cell responses and glaucomatous neurodegeneration and suggesting the crucial involvement of adaptive immunity in neurodegeneration associated with glaucoma. Although the primary response may be favorable in protecting the eye, the proceeding events that lead to long-lasting activation of glial cells and adaptive immune responses can be destructive. They disrupt the homeostasis of the retina and result in the dysfunction of the immune privilege status of the eye (Figure 1). The mechanisms of immune regulation in glaucoma demonstrate certain patterns which are similar to those seen in autoimmune diseases. The antigens and complex antibodies involved in the activation of immunity response are found in the sera of the patients. Although it remains unclear if the antibody production contributes to a cause or consequence of glaucoma, detection of these antibodies may serve as early diagnostic markers for the disease that may allow for proper and effective treatment prior to the late stage of the disease where progression has already occurred. Through careful examination of factors, including the activation of the glial cells, upregulation of HSPs, and the presence of different lymphocytes, development of new therapeutic treatment methods that aim to restore physiological mechanisms of self-tolerance, as well as an early detection of the disease may be possible. These studies may also have a broad impact on uncovering the pathogenesis of neurodegenerative disorders in the brain and spinal cord.

## Author Contributions

All authors listed have made a substantial, direct and intellectual contribution to the work, and approved it for publication.

### Conflict of Interest

The authors declare that the research was conducted in the absence of any commercial or financial relationships that could be construed as a potential conflict of interest.
